# A Systematic Review and Meta-Analysis of the Success Rate of the Primary Probing in Pediatric Patients with Congenital Nasolacrimal Duct Obstruction in Different Age Groups

**DOI:** 10.3390/medicina61081432

**Published:** 2025-08-08

**Authors:** Zhansaya Sultanbayeva, Auyeskhan Dzhumabekov, Neilya Aldasheva, Botagoz Issergepova, Yerzhan Kuanyshbekov, Maiya Taushanova, Indira Karibayeva

**Affiliations:** 1 Department of Science Management, Kazakh Eye Research Institute, Almaty 050012, Kazakhstan; zh.sultanbaeva@eyeinst.kz (Z.S.); n.aldasheva@eyeinst.kz (N.A.); 2 Department of Science and Consulting, Kazakhstan’s Medical University “KSPH”, Almaty 050060, Kazakhstan; a.dzhumabekov@ksph.kz; 3 Department of Spinal Neurosurgery and Peripheral Nervous System Pathology, National Center for Neurosurgery, Astana 010000, Kazakhstan; kuanyshbekov.yerzhan97@gmail.com; 4 Center for Nursing Excellence, West Kazakhstan Marat Ospanov Medical University, Aktobe 030019, Kazakhstan; dr.taushanova@zkmu.kz; 5 Department of Health Policy and Community Health, Jiann-Ping Hsu College of Public Health, Georgia Southern University, Statesboro, GA 30460, USA; ik01379@georgiasouthern.edu

**Keywords:** congenital nasolacrimal duct obstruction, epiphora, primary probing, systematic review, meta-analysis

## Abstract

*Background:* Primary probing of the nasolacrimal duct remains the first-line surgical intervention for congenital nasolacrimal duct obstruction (CNLDO) in infants and young children. However, age-dependent success rates have been less thoroughly investigated. This systematic review and meta-analysis aims to evaluate the age-related success rates of primary probing in children with CNLDO. *Methods:* Systematic literature searches were performed in PubMed, Web of Science, Scopus, ScienceDirect, and Google Scholar in May 2025. A random-effects model was applied to estimate the overall success rate, while sensitivity analyses and publication bias assessments were performed to explore sources of variability. All statistical analyses were carried out using the “meta” and “metafor” packages in RStudio. *Results:* This meta-analysis reveals age-stratified success rates of primary probing for CNLDO: the highest pooled success rate occurred in infants aged 0–6 months (90.67%, I^2^ = 81%, *p* < 0.01), with procedures under general anesthesia achieving 95.42% (I^2^ = 50%; *p* = 0.11) efficacy. Success rates remained favorable in the 6–12 month group (85.18%, I^2^ = 86%, *p* < 0.01 overall; 89.60% with general anesthesia) but declined progressively thereafter (82.34%, I^2^ = 78%, *p* < 0.01 at 12–24 months). While a modest rebound occurred in the 24–48 month group (85.33%, I^2^ = 69%, *p* < 0.01), the oldest cohort (48+ months) demonstrated markedly reduced efficacy (63.47%, I^2^ = 66%, *p* = 0.05), despite exclusive use of general anesthesia. *Conclusion:* Primary probing yields the most favorable outcomes when conducted before 12 months of age, particularly under general anesthesia. Nonetheless, the overall certainty of evidence is low—mainly due to variability across studies—which should be taken into account in clinical decision-making.

## 1. Introduction

Congenital nasolacrimal duct obstruction (CNLDO) is a frequently encountered pediatric ophthalmic disorder, commonly presenting with chronic epiphora and mucopurulent discharge and caused by a persistent membrane at the valve of Hasner [1]. The condition affects approximately 5–20% of newborns, with a significantly higher incidence observed in premature infants [2]. 

In most cases, CNLDO resolves spontaneously during the first year of life without the need for surgical intervention [3]. However, in a subset of patients with persistent symptoms, surgical management becomes necessary [4]. Primary probing is widely regarded as the initial intervention of choice due to its broad accessibility, minimal anesthesia requirements, and relatively low cost [5].

Primary probing can be performed as a simple blind procedure or under endoscopic guidance, which allows direct visualization of the nasolacrimal outflow system and may improve accuracy in complex cases [6,7]. However, in many specialized pediatric ophthalmology settings, simple primary probing continues to represent the predominant approach for managing CNLDO, owing to its feasibility, lower resource demands, and extensive clinical familiarity among providers [8].

Despite its well-established role in clinical practice, the optimal timing of probing remains a subject of ongoing debate in the literature [9]. Although existing systematic reviews have consistently supported the efficacy of probing over alternative interventions such as silicone intubation or balloon catheter dilation, the impact of patient age at the time of surgery on treatment success has not been thoroughly examined [10,11,12,13,14].

This systematic review and meta-analysis aim to evaluate the success rate of primary probing for CNLDO across different pediatric age groups, with the goal of identifying the optimal window for intervention.

## 2. Materials and Methods

### 2.1. Study Registration

This study protocol has been registered with the International Prospective Register of Systematic Reviews (PROSPERO) under the reference number CRD420251057043 [15].

### 2.2. Search Strategy

An initial search of the PROSPERO database was conducted to identify registered protocols for studies of similar scope; however, no such protocols were found. Subsequently, a comprehensive literature search was performed across PubMed, Web of Science, Science Direct, Scopus, and Google Scholar. The search was conducted through June 2025, with no restrictions on the year of publication. Filters applied included publication in English, inclusion in peer-reviewed journals, and restriction to document types such as articles, research articles, and early access articles. To identify appropriate search terms, a preliminary search was conducted in PubMed to extract relevant keywords from the titles and abstracts of studies focused on probing in congenital nasolacrimal duct obstruction (CNLDO). Based on this preliminary analysis, the final search strategy incorporated the following terms: “probing” OR “primary probing” OR “probing in various ages” AND “congenital nasolacrimal duct obstruction” OR “congenital nasolacrimal duct obstruction in children”. Further details of the search strategy are provided in Supplemental Table S1. 

### 2.3. Eligibility Criteria, Study Selection and Data Collection

Supplemental Table S2 presents the eligibility criteria used to select articles in accordance with the Population, Intervention, Comparator, Outcome, and Study Design (PICOS) framework. The study population comprised pediatric patients diagnosed with CNLDO. Studies involving syndromic CNLDO (e.g., craniofacial anomalies, Down syndrome) were omitted from the analysis. The intervention of interest was primary probing performed for the treatment of CNLDO. Studies were excluded if they involved secondary or repeat probing procedures, alternative surgical interventions, or conservative (non-surgical) management approaches. Studies in which primary probing was performed under endoscopic guidance were also excluded, as were those involving patients with congenital craniofacial or bony anomalies, Down syndrome, or other syndromic conditions. No comparator was applicable. The outcome of interest was the success rate of primary probing, defined as the complete remission of symptoms and signs on follow-up. This includes the absence of epiphora, mucous discharge, and increased tear lake, as well as confirmation through caregiver-reported symptom resolution via questionnaire and objective findings such as a normal result on the dye disappearance test. Studies that focused solely on surgical technique without reporting clinical outcomes were excluded. In addition, studies that investigated anatomical variations, treatment-related complications, or the use of specific anesthetic agents and adjunctive pharmacological therapies during probing—without reporting treatment efficacy—were also excluded. The review included observational studies with cross-sectional, prospective, or retrospective designs. Review articles, abstracts, editorials, commentaries, case reports, and publications in languages other than English were excluded.

The eligibility assessment and data collection were conducted in accordance with the Preferred Reporting Items for Systematic Reviews and Meta-Analyses (PRISMA) guidelines [16]. A standardized search was independently performed by two researchers (Z.S. and I.K.). Following the completion of the database searches, all retrieved records were compiled in Microsoft Excel, and duplicate entries were removed. Thereafter, unique records were screened for relevance based on titles and abstracts. In the final phase of eligibility assessment, full-text articles were reviewed for compliance with the predefined inclusion criteria. Data from eligible studies were extracted using a standardized data collection form. Extracted variables included the last name of the first author, year of publication, country of study, study design, total number of eyes treated, age groups of patients, success rate of the CNLDO primary probing (based on the number of eyes), and the type of anesthesia used. Additionally, data were collected on the definition and measurement of outcomes, the specific method used to assess probing success (e.g., clinical resolution, dye disappearance test, patency testing), and the follow-up period.

The two independently completed datasheets were compared and merged. Any discrepancies in study selection or data extraction were resolved through discussion with a third reviewer (B.I.), and consensus was reached for all included studies.

### 2.4. Meta-Analysis

The meta-analysis of proportions was conducted using RStudio in R (version 4.3.2) [17]. Meta and metafor packages were used to calculate the pooled success rates of primary probing for CNLDO across different age groups, along with 95% confidence intervals. Heterogeneity was quantified using the I^2^ statistic and Cochran’s Q test. A random-effect model was applied as heterogeneity exceeded 50%. Forest plots were generated to present the pooled estimates across five age groups: <6 months, 6–12 months, 12–24 months, 24–48 months, and >48 months. To assess potential moderators influencing treatment success, a meta-regression analysis was conducted using the year of publication as a covariate. To explore the influence of individual studies on overall heterogeneity and effect size, a sensitivity analysis was performed. For age groups with more than ten studies, a risk of bias evaluation was conducted. The risk of publication bias was assessed through visual inspection of funnel plots, supplemented by Egger’s regression test for asymmetry. For the subgroup analyses, studies were categorized according to the type of anesthesia used.

### 2.5. Risk of Bias and Certainty of Evidence

To assess the quality of the cohort studies included in this review, we utilized an adapted version of the Newcastle-Ottawa Scale (NOS), which focuses on three core domains: Selection (four criteria), Comparability (one criterion), and Outcome (three criteria) [17]. Each criterion can earn one point, while Comparability may receive up to two, resulting in a maximum possible score of 9. A higher score reflects stronger methodological validity and lower potential for bias. Two authors (Z.S. and I.K.) independently reviewed each study after aligning their evaluation approach. Any differences in scoring were resolved through inter-rater reliability analysis, conducted by a third author (B.I.). Studies that received a total score of five or above were judged to meet the minimum quality threshold and were included in the final analysis (Supplemental Table S3). The certainty of evidence was assessed using the “GRADE” approach in RStudio, which evaluates five key domains: risk of bias (based on the Newcastle–Ottawa Scale as applied to the included cross-sectional studies), inconsistency (quantified using the I^2^ statistic), indirectness (judged according to PICO alignment), imprecision (determined by whether the 95% confidence interval of the pooled estimate overlapped a clinically relevant threshold), and publication bias (evaluated using Egger’s test).

## 3. Results

### 3.1. Study Selection and Characteristics of the Included Studies

**Figure 1:**
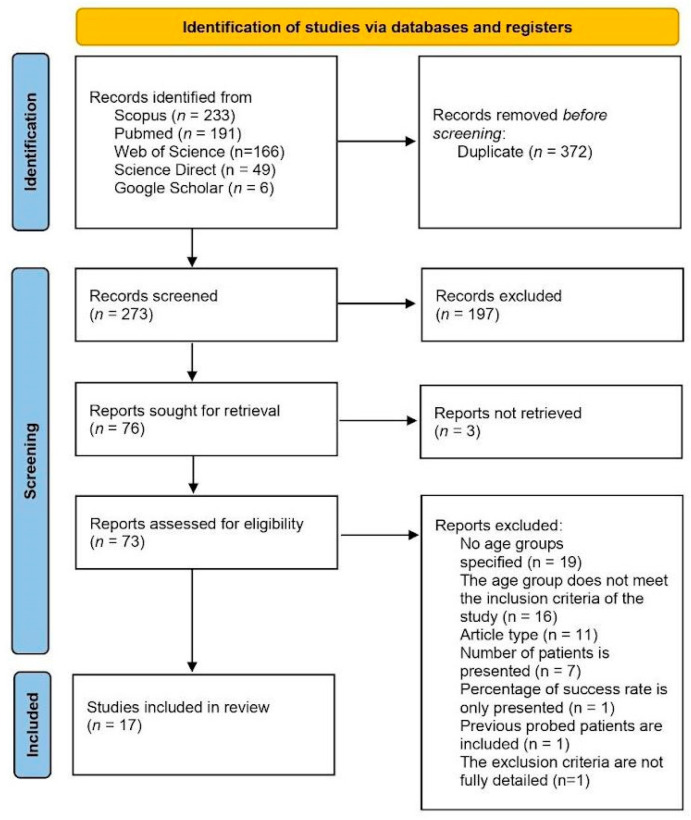
PRISMA flow diagram of study selection.

A total of 645 articles were identified using the search strategy described above. After removing duplicates, 273 titles and abstracts were screened, of which 76 articles were selected for full-text evaluation. However, the full text was unavailable for three studies. Following a full-text assessment, 17 studies met the PICOS eligibility criteria and were included in the meta-analysis. Among the excluded articles, 19 did not specify age groups, and 16 included age groups that did not meet the study’s inclusion criteria. Eleven articles were excluded based on article type. In seven studies, only the number of patients was reported without sufficient outcome data regarding the number of treated eyes. One study reported only the percentage of the success rate without providing raw data [18]. Additionally, one article was excluded for including previously probed patients [19], and another was excluded not defining whether syndromic CNLDO was included or excluded [20]. The PRISMA flowchart illustrating the study selection process is presented in Figure 1.

The included studies, published between 1987 and 2022, were predominantly retrospective cohort studies, with a smaller number of prospective cohorts and comparative series. Most were conducted in the European and South-East Asia World Health Organization (WHO) regions, with the European region being the most represented. Probing was most commonly performed under general anesthesia, although several studies used topical anesthesia, particularly in younger age groups. Follow-up durations ranged from 1 day to 82 months, with most studies reporting follow-up between 2 weeks and 6 months. Collectively, the seventeen studies assessed a total of 7110 eyes, identifying success rate after primary probing in 6059 eyes. Detailed information on the included articles is provided in Table 1.

### 3.2. Meta-Analysis of Primary Probing

**Figure 2:**
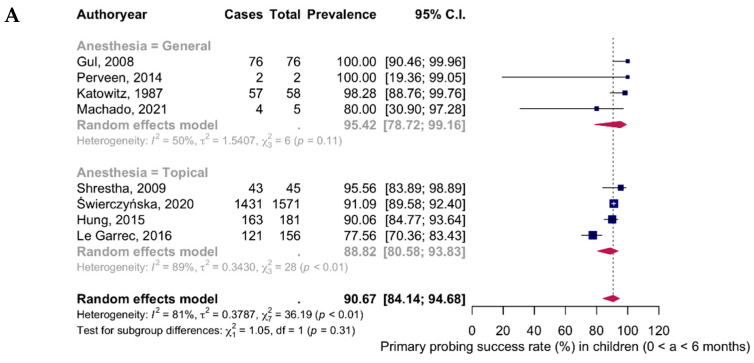

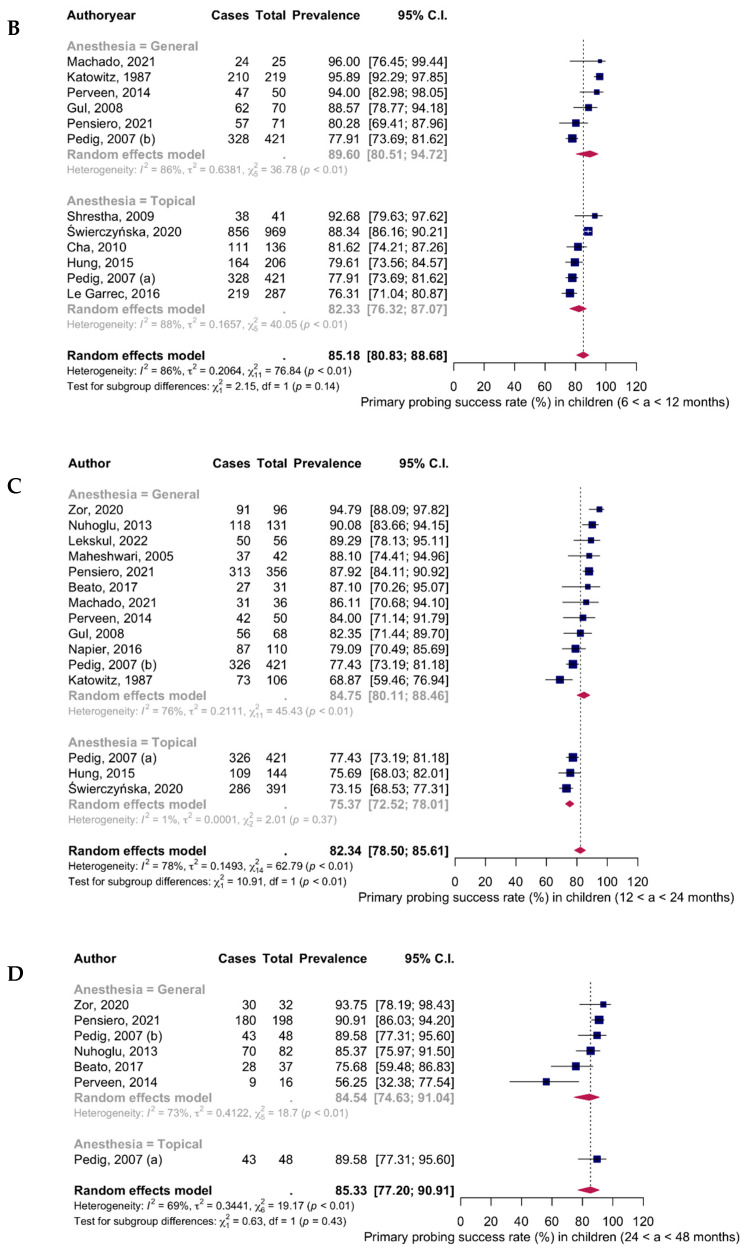

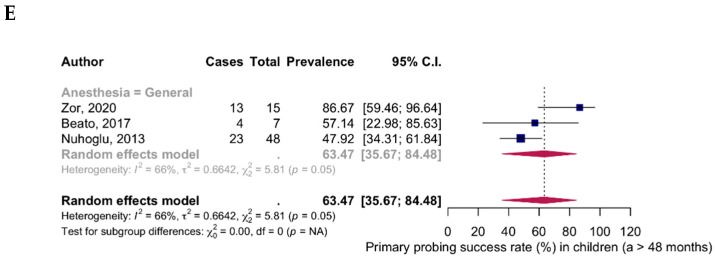
Meta-analysis of the success rate of primary probing in children: (**A**) 0–6 Months; (**B**) 6–12 Months; (**C**) 12–24 Months; (**D**) 24–48 Months; (**E**) >48 Months. Studies included to the meta-analysis: Katowitz, 1987 [21], Maheshwari, 2005 [22], PEDIG, 2007 [23], Gul, 2008 [24], Shrestha, 2009 [25], Cha, 2010 [26], Nuhoglu, 2013 [27], Perveen, 2014 [28], Hung, 2015 [29], Napier, 2016 [30], Le Garrec, 2016 [31], Beato, 2017 [32], Świerczyńska, 2020 [33], Zor, 2020 [34], Machado, 2021 [35], Pensiero, 2021 [36], Lekskul, 2022 [37]. Abbreviations: CI—confidence interval. Pedig, 2007 (a)—topical anesthesia; Pedig, 2007 (b)—general anesthesia.

The results of the meta-analysis assessing the success rate of primary nasolacrimal duct probing in children, stratified by age and type of anesthesia, are presented in Figure 2 and summarized in Table 2. Panel A presents data for children aged 0 to 6 months. Total number of eyes assessed was 2094 and the total number of success rate was 1897. The overall pooled success rate, calculated using a random-effects model, was 90.67% (95% CI: 84.14–94.68; I^2^ = 81%; *p* < 0.01), indicating substantial heterogeneity. In cases where general anesthesia was used, the pooled success rate was higher at 95.42% (95% CI: 78.72–99.16; I^2^ = 50%; *p* = 0.11), reflecting moderate heterogeneity. In contrast, cases using topical anesthesia had a pooled success rate of 88.82% (95% CI: 80.58–93.83; I^2^ = 89%; *p* < 0.01), indicating high heterogeneity. The lowest reported success rate was 77.56% [31], while the highest (100%) was reported by Perveen (2014) [28] and Gul (2008) [24].

Panel B presents data for children aged 6 to 12 months. Total number of eyes assessed was 2495, and the total number of success rate was 2116. The overall pooled success rate was 85.18% (95% CI: 80.83–88.68; I^2^ = 86%; *p* < 0.01), indicating substantial heterogeneity. Under general anesthesia, the pooled success rate was 89.60% (95% CI: 80.51–94.72; I^2^ = 86%; *p* < 0.01), while under topical anesthesia it was 82.33% (95% CI: 76.32–87.07; I^2^ = 88%; *p* < 0.01), both showing high heterogeneity. The lowest success rate was 76.31% (Le Garrec, 2016) [31], and the highest was 96.00% (Machado, 2021) [35].

Panel C displays data for children aged 12 to 24 months. Total number of eyes assessed was 2038, and the total number of success rate was 1646. The overall pooled success rate was 82.34% (95% CI: 78.50–85.61; I^2^ = 78%; *p* < 0.01), indicating high heterogeneity. In the general anesthesia group, the pooled success rate was 84.75% (95% CI: 80.11–88.46; I^2^ = 76%; *p* < 0.01), while in the topical anesthesia group it was 75.37% (95% CI: 72.52–78.01; I^2^ = 1%; *p* = 0.01), indicating no meaningful heterogeneity. The lowest rate (68.87%) was reported by Katowitz (1987) [21], and the highest (94.79%) by Zor (2020) [34], both under general anesthesia.

Panel D presents data for children aged 24 to 48 months. Total number of eyes assessed was 413, and the total number of success rate was 360. The overall pooled success rate was 85.33% (95% CI: 77.20–90.91; I^2^ = 69%; *p* < 0.01), suggesting high heterogeneity. Among the seven studies in this subgroup, six used general anesthesia. The pooled success rate for these was 84.54% (95% CI: 74.63–91.04; I^2^ = 73%; *p* < 0.01), indicating high heterogeneity. One study using topical anesthesia reported the highest rate in this age group: 89.58% (95% CI: 77.31–95.60). The lowest success rate (56.25%) was reported by Perveen (2014) [28].

Panel E displays data for children aged 48 months and older. Total number of eyes assessed was 70, and the total number of success rate was 40. The overall pooled success rate was 63.47% (95% CI: 35.67–84.48; I^2^ = 66%; *p* = 0.05), indicating moderate to substantial heterogeneity. All three studies in this group involved procedures under general anesthesia. The lowest reported rate was 47.92% (Nuhoglu, 2013) [27], and the highest was reported by Zor (2020) [34].

**Figure 3:**
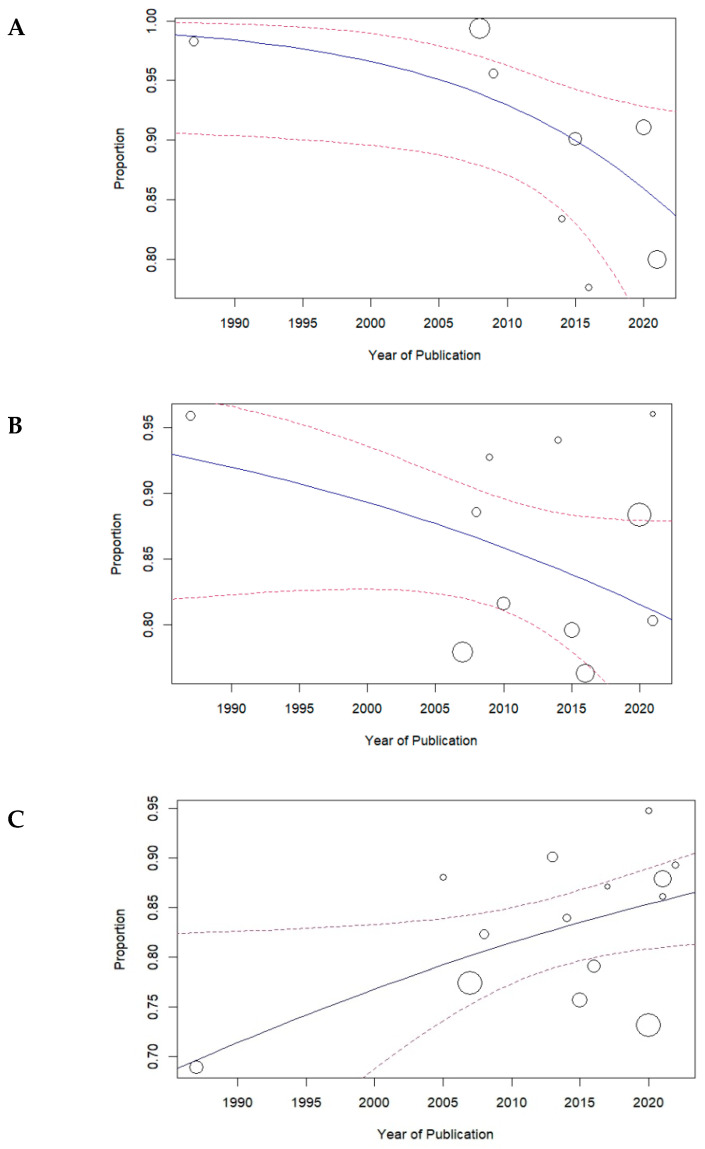

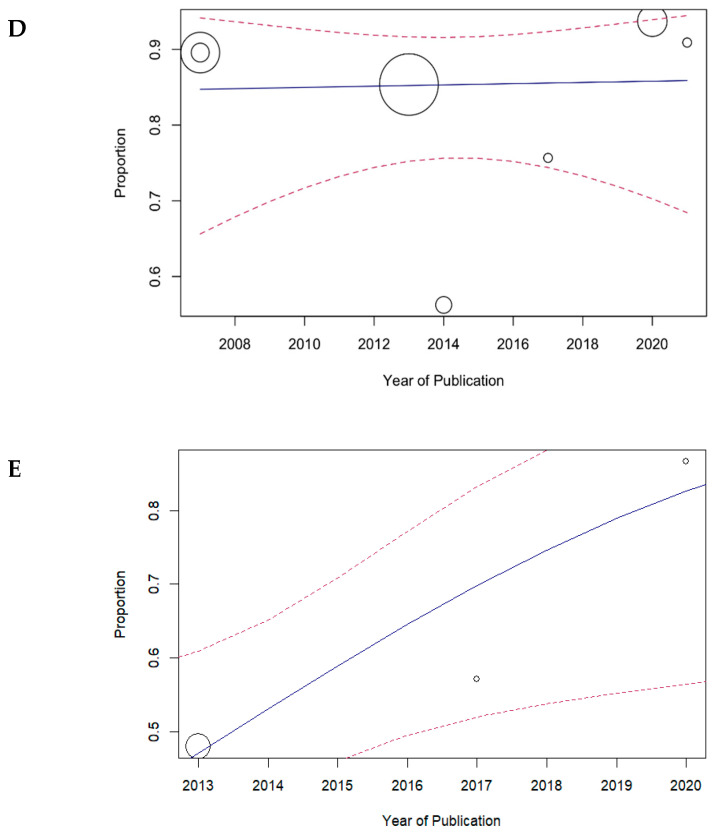
Meta-regression of the success rate of primary probing by year of publication in children: (**A**) 0–6 Months; (**B**) 6–12 Months; (**C**) 12–24 Months; (**D**) 24–48 Months; (**E**) >48 Months.

The results of the meta-regression analysis evaluating the association between the year of publication and the proportion of successful primary probing in children is presented in Figure 3. Panel A presents data for children aged 0 to 6 months. The analysis revealed a statistically significant negative trend over time, with earlier studies reporting higher success rates (*p* = 0.04). The fitted regression line demonstrates a gradual decline in proportions as publication year increases. The widening 95% confidence intervals in more recent years suggest greater heterogeneity or reduced certainty in the estimated proportions, highlighting variability among newer studies.

Panel B presents data for children aged 6 to 12 months. The analysis a downward trend in the association between the year of publication and the proportion of successful primary probing in children in this age group, but this relationship was not significant (*p* = 0.12).

Panel C presents data for children aged 12 to 24 months. The analysis revealed a statistically significant upward trend over time, with recent studies reporting higher success rates (*p* = 0.03).

Panel D presents data for children aged 24 to 48 months. The analysis revealed no association between the year of publication and the proportion of successful primary probing in children in this age group (*p* = 0.91).

Panel E presents data for children aged 48 months and above. The analysis revealed a statistically significant upward trend over time, with recent studies reporting higher success rates (*p* = 0.02).

**Figure 4:**
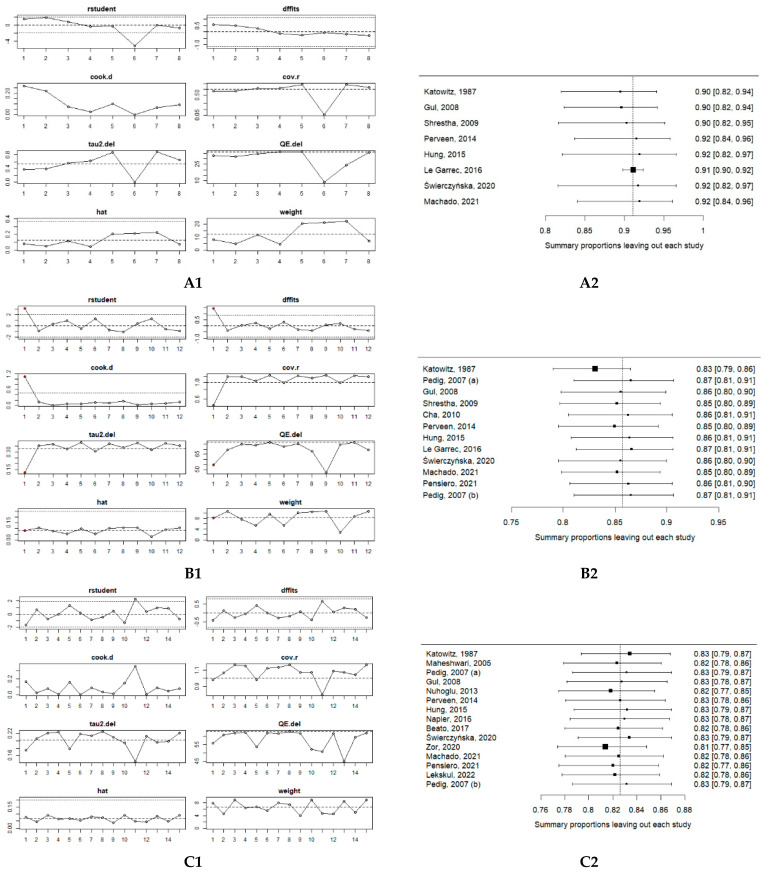

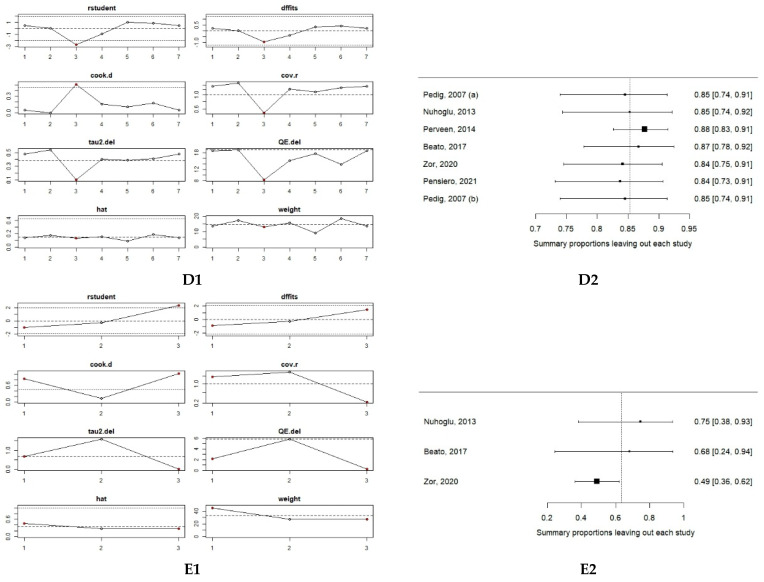
Sensitivity analysis of the success rate of primary probing in children: (**A1**) influence analysis 0–6 Months; (**B1**) influence analysis 6–12 months; (**C1**) influence analysis 12–24 months; (**D1**) influence analysis 24–48 months; (**E1**) influence analysis > 48 months; (**A2**) leave-one-out analysis 0–6 months; (**B2**) leave-one-out analysis 6–12 months; (**C2**) leave-one-out analysis 12–24 months; (**D2**) leave-one-out analysis 24–48 months; (**E2**) leave-one-out analysis > 48 months. Abbreviations: CI—confidence interval. Pedig, 2007 (a)—topical anesthesia; Pedig, 2007 (b)—general anesthesia. Included studies of the sensitivity analysis: Katowitz, 1987 [21], Maheshwari, 2005 [22], PEDIG, 2007 [23], Gul, 2008 [24], Shrestha, 2009 [25], Cha, 2010 [26], Nuhoglu, 2013 [27], Perveen, 2014 [28], Hung, 2015 [29], Napier, 2016 [30], Le Garrec, 2016 [31], Beato, 2017 [32], Świerczyńska, 2020 [33], Zor, 2020 [34], Machado, 2021 [35], Pensiero, 2021 [36], Lekskul, 2022 [37].

Sensitivity analyses—leave-one-out and influence diagnostics—were undertaken to test the robustness of the pooled estimates. As illustrated in Figure 4 (Panel A1), no study exerted the strongest influence on the pooled success rate for primary nasolacrimal duct probing in children aged 0–6 months. Excluding the study by Le Garrec, 2016 [31] raised the pooled estimate to 0.91 and tightened the 95% CI to 0.90; 0.92, yet it did not significantly influence the overall result (Figure 4, Panel A2).

The influence diagnostics of the studies on children aged 6–12 months revealed that Katowitz, 1987 [21] was an influential study (pooled average: 95.89% (95% CI: 92.29; 97.85), as shown in Figure 4 (Panels B1). Excluding this study decreased the pooled estimate to 0.83 (95% CI: 0.80–0.86), indicating that the overall result was sensitive to this study results, as shown in Figure 4 (Panels B2).

The influence analysis for studies on children aged 12–24 months showed that no individual study had a strong effect on the overall findings Figure 4 (Panel C1). The leave-one-out analysis showed that none of the studies had a significant influence on the overall result, confirming its stability Figure 4 (Panel C2).

The influence diagnostics of the studies on children aged 24–48 months revealed that Perveen, 2014 [28] was an influential study (pooled average: 56.25% (95% CI: 32.38; 77.54), as shown in Figure 4 (Panel D1). The leave-one-out analysis showed that excluding this study increased the pooled estimate to 0.88 (95% CI: 0.83; 0.91), indicating that the overall result was sensitive to this study results, as shown in Figure 4 (Panels D2).

The influence diagnostics of the studies on children aged 48 months and above revealed that Nuhoglu, 2013 [27] 47.92% (95% CI 34.31; 61.84) and Zor, 2020 [34] 86.67% (95% CI: 59.46; 96.64) both were influential studies, as shown in Figure 4 (Panel E1). The leave-one-out analysis showed that excluding Nuhoglu, 2013 [27] study increased the pooled estimate to 0.75 (95% CI 0.38–0.93), whereas excluding Zor, 2020 [34] study reduced it to 0.49 (95% CI 0.36–0.62), demonstrating that the pooled result for this oldest subgroup is highly sensitive to these two studies, as shown in Figure 4 (Panels E2).

**Figure 5:**
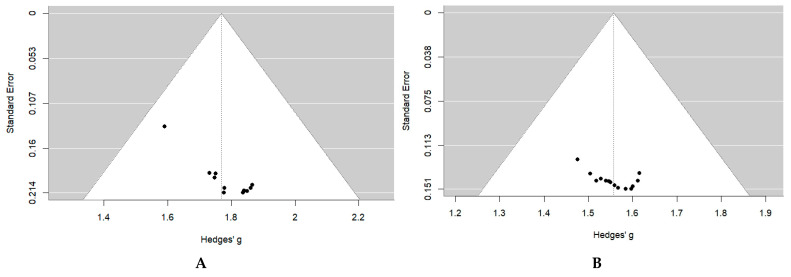
Publication bias assessment of the meta-analysis of the success rate of primary probing in children: (**A**) 6–12 months; (**B**) 12–24 months.

Publication bias was assessed only for pooled estimates that included more than ten studies. For studies on children aged 6–12 months, the funnel plot was visually symmetrical, with points evenly distributed around the pooled effect size, suggesting no risk of bias. Egger’s test likewise showed no significant asymmetry (z = 1.56, *p* = 0.12), confirming the absence of publication bias (Figure 5, Panel A).

A similar pattern emerged for the studies on children aged 12–24 months. The funnel plot was broadly symmetrical, and Egger’s test detected no significant asymmetry (z = 0.74, *p* = 0.46). Together, these findings indicate that publication bias is not present in the studies for this age group (Figure 5, Panel B).

Certainty of evidence assessment results for all pooled estimates presented as the results of the present systematic review and meta-analysis are presented in Table 3. All five meta-analytic outcomes were assessed from the observational studies. The risk of bias was low in all age groups. The assessment shows that inconsistency was rated as “serious” in all age subgroups. Indirectness and imprecision were rated as “not serious” in all age groups. Publication bias was assessed in two age groups out of five, and in both cases it was not detected. Overall, the certainty of the evidence was judged as “low” in all subgroups.

## 4. Discussion

In this systematic review and meta-analysis, we evaluated the success rates of primary probing for CNLDO across distinct pediatric age brackets to determine the optimal timing for intervention. Based on the analyzed data, the 0–6 months age group demonstrated the highest overall success rate of primary probing at 90.67%. Within this group, procedures performed under general anesthesia yielded a significantly higher success rate (95.42%) compared to those performed under topical anesthesia (88.82%). In the 6–12 months group, the success rate decreased to 85.18%, although probing under general anesthesia in this group showed outcomes nearly comparable to the overall result of the 0–6 months group. In the 12–24 months group, the success rate further declined to 82.34%, with general anesthesia being more commonly used than topical anesthesia. Interestingly, in the 24–48 months group, the success rate slightly increased again to 85.33%, with a higher proportion of cases performed under topical anesthesia (89.58%) compared to general anesthesia (84.54%). In the 48+ months group, the success rate dropped to 63.47%, with all procedures conducted under general anesthesia. Despite the observed heterogeneity, the highest success rates were recorded when primary probing was performed within the first six months of life, followed by the 6–12 month period—especially when general anesthesia was used. Furthermore, probing under general anesthesia remains preferable up to 24 months of age to maximize success rates.

The results of our meta-analysis are consistent with the prevailing recommendations and published literature regarding the timing of primary probing in children with CNLDO. Most clinical guidelines, including those from the Pediatric Eye Disease Investigator Group (PEDIG) and the American Academy of Ophthalmology (AAO), recommend performing primary probing between 6 and 15 months of age [5,38]. This timing seeks to balance the high likelihood of spontaneous resolution during early infancy with the declining efficacy of probing observed in older children. Our findings are broadly consistent with these recommendations, demonstrating high pooled success rates in both the 0–6 month (90.67%) and 6–12 month (85.18%) age groups—particularly when general anesthesia is used. In a recent meta-analysis by Farat et al., probing performed between 6 and 10 months of age demonstrated a comparable success rate to that performed between 12 and 16 months, although the certainty of the evidence was rated as low [12]. This raises the possibility that the procedural efficacy of probing remains relatively stable across this broader age range. Taken together, these data suggest that while early probing (<6 months) has traditionally been reserved for complicated or refractory cases of congenital nasolacrimal duct obstruction (CNLDO), it may be underutilized in otherwise uncomplicated yet persistent cases. The consistently high success rates observed in younger infants imply that earlier intervention could not only shorten the duration of symptoms but also reduce caregiver burden and the need for more complex or repeated procedures later in childhood [7]. In children aged 12–24 months and 24–48 months, we observed a gradual decline in the pooled success rates of primary probing (82.34% and 85.33%, respectively). These findings are consistent with international data: for example, the AAO EyeNet reports success rates decreasing from 83.1% at 12–18 months to 71.4% at 18–24 months, and further to 64.7% in children older than 24 months [5]. These patterns support the notion that delayed intervention may be associated with reduced procedural success, reinforcing the importance of timely management in cases of persistent CNLDO. Finally, in children aged 48 months and older, success rates dropped significantly (63.47%), in line with the literature, which suggests decreased efficacy of primary probing beyond this age and recommends alternative treatments such as silicone intubation or dacryocystorhinostomy [12]. In clinical practice, decisions regarding the timing and setting of probing should incorporate shared decision-making, particularly in borderline cases. Counseling should consider not only the age-related success rates, but also the child’s health status, family preferences, anesthesia-related risks, and resource availability to ensure patient-centered care.

From a practical and economic standpoint, primary probing for CNLDO—particularly when performed before the age of 12 months—can be considered a cost-efficient option. Several studies have reported that early intervention significantly reduces the likelihood of requiring more complex and expensive procedures, such as silicone intubation or dacryocystorhinostomy, which are generally indicated in older children or in cases resistant to initial treatment [39,40]. When probing is successful at an earlier age, it often eliminates the need for repeat interventions, minimizes follow-up visits, and lowers the indirect costs associated with extended caregiving, missed workdays, and the psychological burden on families [13]. A cost-effectiveness analysis showed that an approach that reserves conservative management for infants up to five months old and employs probing for those older than five months offers the greatest cost-to-effectiveness value. These observations highlight that early primary probing may not only improve clinical outcomes but also represent a rational and economically sound approach, especially in resource-constrained healthcare settings. 

From an economic perspective, the use of general anesthesia for early primary probing may initially appear contradictory to cost-saving strategies. However, evidence suggests that early intervention—even when performed under general anesthesia—can be both clinically effective and economically justified. For instance, the Pediatric Eye Disease Investigator Group (PEDIG) conducted a randomized trial comparing immediate office-based probing with deferred facility-based probing and found that early probing led to higher success rates (92% vs. 82%) and lower overall costs ($562 vs. $701) [41]. Although office-based probing is typically less expensive, general anesthesia allows for better control and higher success, particularly in uncooperative infants, thereby reducing the need for repeat procedures and minimizing long-term healthcare and societal costs. These findings support the notion that the upfront investment in general anesthesia may be offset by improved treatment efficiency and reduced downstream expenditures—an important consideration in both high- and low-resource settings.

Overall, our findings validate the current evidence base while also suggesting that probing before 12 months—particularly under general anesthesia—may offer excellent outcomes in appropriately selected cases. These insights may inform clinical decision-making and help refine the optimal timing for intervention in pediatric CNLDO. Notably, Kazakhstan currently lacks national guidelines for CNLDO management. As most authors of this study are based in Kazakhstan, our results could inform evidence-based criteria for determining the timing of primary probing in local practice.

### 4.1. Limitations

All included studies were simple observational cohorts without randomization, introducing potential selection bias and limiting the overall strength of the evidence. While our analysis focused on age-stratified outcomes, we were unable to account for clinical complexity—such as simple versus complex CNLDO—due to inconsistent or absent reporting of these classifications in the original studies. As a result, the possible influence of anatomical variability or severity of obstruction on probing outcomes could not be reliably evaluated. Heterogeneity across studies remained moderate to high in several age-based subgroups. To address this, we stratified the data by type of anesthesia (general vs. topical), which partially reduced heterogeneity and allowed for a more nuanced interpretation of pooled success rates. Residual heterogeneity is likely attributable to unexamined factors such as differences in probing technique, postoperative assessment methods, and follow-up duration. Additionally, the limited number of studies in older age groups—particularly those involving children over 48 months—restricts the generalizability of findings in these subpopulations. Given that all included studies were observational in nature and exhibited varying degrees of heterogeneity, our findings should be interpreted with caution when determining the optimal age for probing. These limitations underscore the need for more uniform study designs and reporting standards to strengthen future evidence. Furthermore, while this meta-analysis sought to evaluate age-related success rates for primary probing, it does not provide sufficient evidence to determine whether certain clinical subgroups might benefit from delayed intervention. This gap highlights an important limitation and suggests a need for future prospective research that stratifies patients based on age, clinical severity, symptom duration, and spontaneous resolution likelihood to better understand optimal timing strategies, and to justify the delayed probing in certain subgroups. Finally, although endoscopic probing has demonstrated favorable outcomes, particularly in older pediatric patients, this technique was not included in the present meta-analysis [42]. Our rationale for this exclusion was based on the intention to evaluate the effectiveness of simple primary probing, which remains the most widely practiced and accessible intervention for CNLDO, especially in regional and resource-limited settings. Simple probing can be performed both in inpatient and outpatient environments and does not require specialized equipment or extensive training. By focusing on this conventional approach, we aimed to provide findings that are broadly applicable to general pediatric ophthalmic practice. Nevertheless, we acknowledge the growing relevance of endoscopic techniques and recommend that future comparative studies further explore their role in optimizing outcomes across different age groups. Despite these constraints, the consistency of the trends observed across age groups provides valuable insight into the age-related efficacy of primary probing.

### 4.2. Future Research Directions

To address these limitations and enhance evidence-based clinical decision-making, future research should aim to perform the following:

- Conduct larger, prospective, multicenter randomized controlled trials stratified by age, type of anesthesia (general vs. topical), and clinical subtype (simple vs. complex CNLDO) to provide more robust and generalizable data on probing outcomes.
- Investigate long-term outcomes, including recurrence rates, need for secondary procedures, and quality-of-life measures in children undergoing primary probing at various ages.
- Support the development of national or regional clinical practice guidelines in countries where standardized recommendations for the management of CNLDO are currently lacking. Such guidelines would help unify treatment approaches and improve patient outcomes across different healthcare settings.

## 5. Conclusions

This meta-analysis demonstrates that primary probing for CNLDO achieves optimal outcomes in infants aged 0–6 months (overall success rate: 90.67%), with procedures performed under general anesthesia showing particularly high efficacy (95.42%). Similarly, in the 6–12 month age group, the overall success rate was 85.18%, rising to 89.60% when general anesthesia was utilized. However, due to inconsistency among studies, the certainty of evidence remains low across all subgroups and should be considered when applying these results in clinical practice.

## Figures and Tables

**Table 1:** Description of the included studies.

Last Name, Year	Design	Country/WHO Region	Age Group Range (in Months)	Total Eyes Treated	Success Rate After Primary Probing (Eyes)	Type of Anesthesia	Postoperative Evaluation Measures	Follow-Up Period
Katowitz, 1987 [21]	Retrospective cohort study	USA/America	0 < a < 6 6 < a < 12 12 < a < 24	58 219 106	57 210 73	General	Observation DDT	1 week–6 months
Maheshwari, 2005 [22]	Retrospective cohort study	India/South-East Asia	12 < a < 24	42	37	General	CRSS	1 week–3 months
PEDIG, 2007 [23]	Prospective cohort study	USA/America	6 < a< 12 12 < a < 24 24 < a < 48	421 421 48	328 326 43	General/ Topical	CRSS The questionnaire DDT	1 month ± 1 week
Gul, 2008 [24]	Prospective cohort study	Pakistan/Eastern Mediterranean	0 < a < 6 6 < a < 12 12 < a < 24	76 70 68	76 62 56	General	CRSS	1 week–3 months
Shrestha, 2009 [25]	Prospective cohort study	Nepal/South-East Asia	0 < a < 6 6 < a < 12	45 41	43 38	Topical	CRSS	3–6 weeks
Cha, 2010 [26]	Retrospective comparative case series	South Korea/Western Pacific	6 < a < 12	136	111	Topical	CRSS	1 week–2 months
Nuhoglu, 2013 [27]	Retrospective cohort study	Turkey/European	12 < a < 24 24 < a < 48 a > 48	131 82 48	118 70 23	General	The questionnaire DDT	1 day–3 months
Perveen, 2014 [28]	Prospective cohort study	India/South-East Asia	0 < a < 6 6 < a < 12 12 < a < 24 24 < a < 48	2 50 50 16	2 47 42 9	General	CRSS	2 weeks–6 months
Hung, 2015 [29]	Prospective cohort study	Taiwan/Western Pacific	0 < a < 6 6 < a< 12 12 < a < 24	181 206 144	163 164 109	Topical	CRSS	1 week
Napier, 2016 [30]	Retrospective comparative study	United Kingdom/European	12 < a < 24	110	87	General	CRSS	6–12 weeks
Le Garrec, 2016 [31]	Retrospective cohort study	France/European	0 < a < 6 6 < a < 12	156 287	156 287	Topical	CRSS	4 weeks
Beato, 2017 [32]	Retrospective cohort study	Portugal/European	12 < a < 24 24 < a < 48 a > 48	31 37 7	27 28 4	General	CRSS	1–82 months
Świerczyńska, 2020 [33]	Retrospective cohort study	Poland/European	0 < a < 6 6 < a < 12 12 < a < 24	1571 969 391	1431 856 286	Topical	CRSS DDT	3 weeks–6 months
Zor, 2020 [34]	Retrospective cohort study	Turkey/Europaen	12 < a < 24 24 < a < 48 a > 48	96 32 15	91 30 13	General	CRSS DDT	6–36 months
Machado, 2021 [35]	Retrospective cohort study	Brazil/America	0 < a < 6 6 < a < 12 12 < a < 24	5 25 36	4 24 31	General	CRSS The questionnaire DDT	4, 81 months
Pensiero, 2021 [36]	Retrospective cohort study	Italy/European	6 < a < 12 12 < a < 24 24 < a < 48	71 356 198	57 313 180	General	CRSS	6 months
Lekskul, 2022 [37]	Retrospective cohort study	Thailand/South-East Asia	12 < a < 24	56	50	General	CRSS	1 month

Abbreviations: DDT—dye disappearance test; CRSS—complete remission of symptoms and signs; PEDIG—Pediatric Eye Disease Investigator Group.

**Table 2:** Summary table of key findings of the meta-analysis.

Age Group	Eyes Assessed (*n*)	Successful Cases (*n*)	Pooled Mean Success Rate (%, 95% CI, I^2^)
0–6 months (8 studies)			
General anesthesia	141	139	95.42% (95% CI: 78.72–99.16; I^2^ = 50%)
Topical anesthesia	1953	1758	88.82% (95% CI: 80.58–93.83; I^2^ = 89%)
Total	2094	1897	90.67% (95% CI: 84.14–94.68; I^2^ = 81%)
6–12 months (11 studies)			
General anesthesia	856	728	89.60% (95% CI: 80.51–94.72; I^2^ = 86%)
Topical anesthesia	2060	1716	82.33% (95% CI: 76.32–87.07; I^2^ = 88%)
Total	2495	2116	85.18% (95% CI: 80.83–88.68; I^2^ = 86%)
12–24 months (14 studies)			
General anesthesia	1503	1251	84.75% (95% CI: 80.11–88.46; I^2^ = 76%)
Topical anesthesia	956	721	75.37% (95% CI: 72.52–78.01; I^2^ = 1%)
Total	2038	1646	82.34% (95% CI: 78.50–85.61; I^2^ = 78%)
24–48 months (6 studies)			
General anesthesia	413	360	84.54% (95% CI: 74.63–91.04; I^2^ = 73%)
Topical anesthesia	48	43	89.58% (95% CI: 77.31–95.60)
Total	413	360	85.33% (95% CI: 77.20–90.91; I^2^ = 69%)
>48 months (3 studies)			
General anesthesia	70	40	63.47% (95% CI: 35.67–84.48; I^2^ = 66%)
Topical anesthesia	0	0	
Total	70	40	63.47% (95% CI: 35.67–84.48; I^2^ = 66%)

**Table 3:** Evaluation of the certainty of evidence using GRADE framework on the success rate of primary probing in children.

Outcome	Study Design	Risk of Bias	Inconsistency	Indirectness	Imprecision	Publication Bias	Certainty of Evidence
Pooled success rate of nasolacrimal duct probing (0–6 months)	Meta-analysis of observational studies	Low	Serious	Not serious	Not serious	Not assessed (*n* < 10)	Low
Pooled success rate of nasolacrimal duct probing (6–12 months)	Meta-analysis of observational studies	Low	Serious	Not serious	Not serious	Not detected	Low
Pooled success rate of nasolacrimal duct probing (12–24 months)	Meta-analysis of observational studies	Low	Serious	Not serious	Not serious	Not detected	Low
Pooled success rate of nasolacrimal duct probing (24–48 months)	Meta-analysis of observational studies	Low	Serious	Not serious	Not serious	Not assessed (*n* < 10)	Low
Pooled success rate of nasolacrimal duct probing (≥48 months)	Meta-analysis of observational studies	Low	Serious	Not serious	Serious	Not assessed (*n* < 10)	Low

## Data Availability

The original contributions presented in this study are included in the article. Further inquiries can be directed to the corresponding author.

## Supplementary Materials

The following supporting information can be downloaded at: https://www.mdpi.com/article/10.3390/medicina61081432/s1, Table S1: Search strategy for the systematic review; Table S2: Inclusion and exclusion criteria of study selection based on the PICOS framework; Table S3: Risk of Bias (Quality) Assessment according to the New-Castle Ottawa Scale [21,22,23,24,25,26,27,28,29,30,31,32,33,34,35,36,37].

## Abbreviations

The following abbreviations are used in this manuscript:

CNLO	Congenital Nasolacrimal Duct Obstruction
PRISA	Preferred Reporting Items for Systematic Reviews and Meta-Analyses
NOS	Newcastle-Ottawa Scale
WHO	World Health Organization
DDT	Dye disappearance test
CRSS	Complete remission of symptoms and signs
PEDIG	Pediatric Eye Disease Investigator Group
CI	Confidence interval
