# Supplementary material for: A Systematic Review and Meta-Analysis of the Success Rate of the Primary Probing in Pediatric Patients with Congenital Nasolacrimal Duct Obstruction in Different Age Groups
Source: Medicina (Kaunas). 2025 Aug 8;61(8):1432. doi: 10.3390/medicina61081432 (PMC12388011; doi:10.3390/medicina61081432)
Supplement: Supplementary file 1 [file medicina-61-01432-s001.zip › Supplemental Table S1.pdf]

Supplemental Table S1. Search strategy for the systematic review.

Database	Search Fields	Filters
PubMed	Title, Abstract, Keywords	Language: English<br>Species: Human
Science Direct	Title, Abstract, Keywords	Language: English
Scopus	Title, Abstract, Keywords	Language: English<br>Document Type: Articles
Web of Science	Title, Abstract, Keywords	Language: English<br>Document Type: Articles
Google Scholar	Title	Language: English
