# Supplementary material for: A Systematic Review and Meta-Analysis of the Success Rate of the Primary Probing in Pediatric Patients with Congenital Nasolacrimal Duct Obstruction in Different Age Groups
Source: Medicina (Kaunas). 2025 Aug 8;61(8):1432. doi: 10.3390/medicina61081432 (PMC12388011; doi:10.3390/medicina61081432)
Supplement: Supplementary file 1 [file medicina-61-01432-s001.zip › Supplemental Table S2.pdf]

Supplemental Table S2. Inclusion and exclusion criteria of study selection based on the PICOS framework.

PICOS framework	Inclusion criteria	Exclusion criteria
Population	Studies that enrolled pediatric patients with CNLDO	Studies that involved pediatric patients diagnosed with CNLDO associated with congenital craniofacial or bony anomalies, Down syndrome, or other syndromic conditions
Intervention	Studies, where primary probing performed for the treatment of CNLDO	Studies included secondary or repeat probing, probing performed under endoscopic guidance, alternative surgical interventions, and non-surgical management
Comparator	Not applicable	Not applicable
Outcome	Studies with the success rate of primary probing, defined as the complete remission of symptoms and signs on follow-up.	Studies that did not report treatment outcomes; studies focusing solely on complications, anatomical variations, or the use of anesthetic agents and adjunctive medications without outcome data
Study design	Observational studies with cross-sectional, prospective, or retrospective designs	Review articles, abstracts, editorials, commentaries, case reports, and studies published in languages other than English
