# Supplementary material for: A Systematic Review and Meta-Analysis of the Success Rate of the Primary Probing in Pediatric Patients with Congenital Nasolacrimal Duct Obstruction in Different Age Groups
Source: Medicina (Kaunas). 2025 Aug 8;61(8):1432. doi: 10.3390/medicina61081432 (PMC12388011; doi:10.3390/medicina61081432)
Supplement: Supplementary file 1 [file medicina-61-01432-s001.zip › Supplemental Table S3.pdf]

Supplemental Table S3. Risk of Bias (Quality) Assessment according to the New-Castle Ottawa Scale

Study	Selection	Comparability	Exposure	Total
Katowitz et al. [21]	3	0	3	6
Maheshwari et al. [22]	3	0	3	6
PEDIG [23]	3	0	3	6
Gul et al. [24]	3	0	3	6
Shrestha et al. [25]	3	0	3	6
Cha et al. [26]	4	1	3	8
Nuhoglu et al. [27]	3	0	3	6
Perveen et al. [28]	3	0	3	6
Hung et al. [29]	3	0	3	6
Napier et al. [30]	4	1	3	8
Le Garrec et al. [31]	3	0	3	6
Beato et al. [32]	3	0	3	6
Świerczyńska et al. [33]	3	0	3	6
Zor et al. [34]	3	0	3	6
Machado et al. [35]	3	0	3	6
Pensiero et al. [36]	3	0	3	6
Lekskul et al. [37]	3	0	3	6
